# Typhoid Due to Laboratory Inoculation

**Published:** 1918-08

**Authors:** Andy Hall

**Affiliations:** Mt. Vernon, Ill.


### Typhoid Due to Laboratory Inoculation.

Dr. ANDY HALL, Mt. Vernon, Ill., Capt. M. R. C.

A colleague of the writer, accustomed to preparing autogenous vaccines and maintaining typhoid cultures, shook a culture tube to distribute the sediment and wet the cotton stopper. He washed his hands with soap and water immediately but used no antiseptic and soon after, rolled and smoked a cigarette. Exactly three weeks later, he noticed the first malaise and found that his temperature was 101. The next day, he felt worse but kept about as usual till later in the day fhen the temperature was 102. He then sought advice and was sent to a hospital. The course of the disease was typic though rather mild. The maximum temperature was 103.5; rose spots appeared, in all about 50 confined to the abdomen; there was no delirium, diarrhoea nor haemorrhage; the subjective symptoms were rather marked, consisting especially of pain in the nape of the neck. Defervescence occurred in three weeks, full recovery in five. Expectant treatment was employed.

An interesting postscript is that, 8 years later, the patient reacted rather severely to triple typhoid inoculation, though this is said to be the rule and the general opinion seems to be that the reaction is due especially to the para-typhoid element.

In this connection may be mentioned the case of another physician who had never had typhoid but who, several years ago received a (non-military) triple typhoid inoculation. A short but rather severe febrile period followed which was pretty definitely explained by a failure to kill all of one of the para-typhoid cultures though the evidence was circumstantial and, naturally, more or less contradicted by statements of the source of the vaccine. This man had an exacerbation of a neuritis immediately following the triple typhoid inoculation. It is obvious that typhoid or para-typhoid from cultures, even if no attempt to kill had been made, would naturally be expected to be somewhat milder than cases derived from ordinary sources of infection, with the virulence of the organism heightened by recent passage through the animal body. A careful collection of laboratory workers' infections might, however, show the a priori view to be incorrect and, even in such a case as the first mentioned, the severity, though not marked, was as great as in many spontaneously infected cases.

